# HiLo Based Line Scanning Temporal Focusing Microscopy for High-Speed, Deep Tissue Imaging

**DOI:** 10.3390/membranes11080634

**Published:** 2021-08-17

**Authors:** Ruheng Shi, Yuanlong Zhang, Tiankuang Zhou, Lingjie Kong

**Affiliations:** 1 State Key Laboratory of Precision Measurement Technology and Instruments, Department of Precision Instrument, Tsinghua University, Beijing 100084, China; srh18@mails.tsinghua.edu.cn; 2 Department of Automation, Tsinghua University, Beijing 100084, China; ylzhang16@mails.tsinghua.edu.cn (Y.Z.); tiankuangzhou@gmail.com (T.Z.); 3 Graduate School at Shenzhen, Tsinghua University, Shenzhen 518055, China; 4 IDG/McGovern Institute for Brain Research, Tsinghua University, Beijing 100084, China

**Keywords:** HiLo microscopy, line scanning temporal focusing microscopy, deep tissue imaging, contrast enhancement, axial confinement enhancement

## Abstract

High-speed, optical-sectioning imaging is highly desired in biomedical studies, as most bio-structures and bio-dynamics are in three-dimensions. Compared to point-scanning techniques, line scanning temporal focusing microscopy (LSTFM) is a promising method that can achieve high temporal resolution while maintaining a deep penetration depth. However, the contrast and axial confinement would still be deteriorated in scattering tissue imaging. Here, we propose a HiLo-based LSTFM, utilizing structured illumination to inhibit the fluorescence background and, thus, enhance the image contrast and axial confinement in deep imaging. We demonstrate the superiority of our method by performing volumetric imaging of neurons and dynamical imaging of microglia in mouse brains in vivo.

## 1. Introduction

Optical-sectioning capability is highly desired in biomedical imaging, as biological cells and tissues are generally in three-dimensions. To achieve optical-sectioning, confocal laser-scanning microscopy was proposed to spatially filter out out-of-focus information with a conjugated pinhole [1,2,3]. In addition, multiphoton microscopy was developed for deep tissue imaging, which achieves optical-sectioning through nonlinear localized excitation and deeper penetration, benefiting from the lesser scattering of longer excitation wavelengths [4,5,6,7]. However, as point-scanning methods, these techniques usually have limitations in temporal resolutions. To solve this problem, spinning-disk confocal microscopy and multifocal two photon microscopy were proposed, which improve the temporal resolution by generating multifocal points through lenslet arrays or spatial light modulators (SLM), which, however, increase the complexity of the microscope setups [8,9,10,11].

Line-scanning two-photon microscopy (LSTPM) can improve temporal resolution by shaping the excitation beam into a thin line, which thus simplifies two-dimensional scanning into one-dimensional scanning. However, the improvement of temporal resolution in LSTPM is at the cost of axial resolution, since the beam is focused only along one spatial dimension [12]. Thus, line scanning temporal focusing microscopy (LSTFM) was developed, which has the capability of parallel excitation while maintaining tight axial confinement by spatio-temporal focusing [13,14,15,16,17,18,19]. However, with the increase in penetration depth, the accumulated wavefront distortions would deteriorate axial confinement of the excitation light. Additionally, scattering of emitted fluorescence would introduce cross-talk, and thus sacrifice the image contrast.

To overcome the effects of tissue scattering on the excitation and/or the emission beams in wide-field two-photon imaging, one can employ either adaptive optics (AO) [19,20,21] or computation imaging. For the former, we demonstrated the hybrid spatio-spectral coherent adaptive compensation for LSTFM [19]. For the latter, we proposed the techniques of focal modulation [22] and extended detection followed by computation reconstructions [18] for LSTFM. Zheng et al. also demonstrated a de-scattering method by taking advantage of patterned nonlinear excitation and computational-assisted wide-field detection [23]. All these methods are effective in enhancing axial resolution and background rejection. However, they are either too complex for implementation, or time-consuming in terms of computational reconstruction.

Structured illumination-based optical-sectioning microscopy was developed to relieve the scattering issues in wide-field imaging, including structured illumination microscopy (SIM-OS) [24,25] and HiLo microscopy [26,27,28,29]. In SIM-OS, three sinusoidal modulated images, each of strictly well-defined phase-shifts, are computationally combined to calculate an optical-sectioning image. However, the sample induced aberration and scattering may distort the modulated patterns, and further result in artifacts in final reconstructed images. In contrast, in HiLo microscopy (“Hi” and “Lo” for the high and low spatial frequency components, respectively), two images (one for uniform illumination image, and one for structured illumination image), are required to reconstruct an optical-sectioning image. Moreover, the effects of distortion of the illumination patterns are not so severe in HiLo method, as it does not need well-defined patterns to modulate in-focus information [30]. However, the temporal resolutions in these techniques are scarified, as more than two images are generally required to reconstruct an optical-sectioning image [30,31,32].

Here, we propose the HiLo-based LSTFM, which not only ensures high temporal resolution with one-time imaging, but also further depresses scattering through HiLo method. Benefiting from the fact that the HiLo method does not need well-defined excitation patterns, we can take the Gaussian intensity distribution of the illumination line as a naturally structured modulation. In this way, HiLo-based LSTFM does not need to take an additional structured illumination image and thus save the temporal resolution. We demonstrate the superiority of our method by volumetric imaging of neurons and dynamical imaging of microglia in mouse brains in vivo.

## 2. Method

### 2.1. Optical Setup

**Figure 1:**
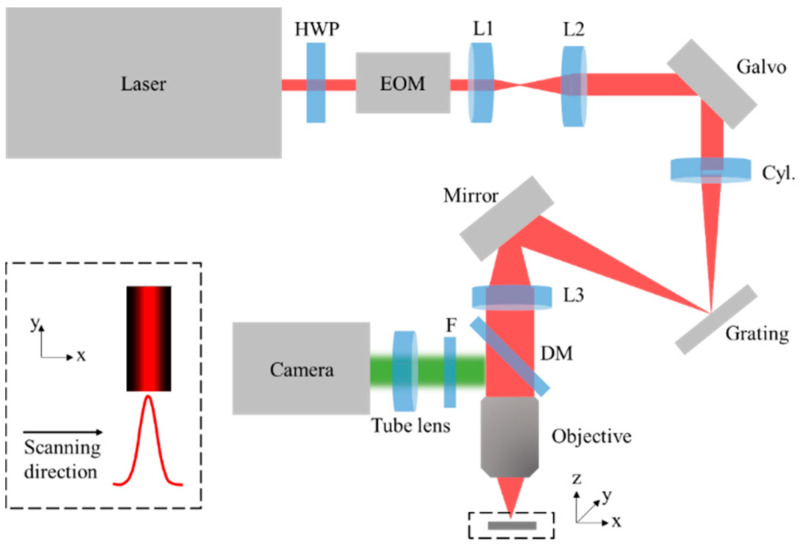
Optical setup of the HiLo-LSTFM. HWP: half wave plate. EOM: electro-optical modulator. L1: lens 1, *f* = 60 mm. L2: lens 2, *f* = 150 mm. Cyl.: cylinder lens, *f* = 300 mm. L3: lens 3, *f* = 200 mm. DM: dichroic mirror. F: filter. Tube lens, *f* = 200 mm. The insert shows that, at the focal plane, the excitation laser is shaped into a spatio-temporal focusing line with Gaussian distribution along the lateral scanning direction.

The optical-setup for HiLo-LSTFM is similar to that in [18]. Briefly, a 920 nm femtosecond laser (80 MHz repetition rate, 120 fs pulse duration, Chameleon Discovery, Coherent) was used for two-photon fluorescence excitation. A half wave plate (HWP) and an electro-optical modulator (EOM, M3202RM, Conoptics, Danbury, CT, USA) were used to control the laser intensity. After passing through a relay set consisting of lens 1 (L1, *f* = 60 mm) and lens 2 (L2, *f* = 150 mm), the laser beam was expended and projected onto the surface of a 1D galvo-scanner (Galvo, S211, Thorlabs, Newton, MA, USA) with a 5-mm diameter. A laser line was then formed on the surface of a diffraction grating (Grating, 830 lines/mm, Edmund Optics, Barrington, IL, USA) after passing through a cylinder lens (Cyl., *f* = 300 mm). The spectral components of the femtosecond pulses were then spatially dispersed due to grating diffraction. After passing through the lens 3 (L3, *f* = 200 mm), dichroic mirror (DM, DMSP750B, Thorlabs, Newton, MA, USA), and objective (XLPLN25XWMP2, 25×, 1.05 NA, water immersion, Olympus, Tokyo, Japan), the spatio-temporal focused line with Gaussian distribution along the scanning direction (insert of Figure 1) was then formed at the focal plane. The emitted fluorescence is collected by the same objective. After passing through a bandpass filter (F, E510/80, Chroma, Bellows Falls, VT, USA) and tube lens (TTL200-A, Thorlabs, Newton, MA, USA), a 2D fluorescence image excited by the Gaussian line was detected with a camera (Zyla 5.5 plus, Andor, Concord, MA, USA), as done in our previous method of extended detection [18]. For 2D imaging, lateral scanning was performed by steering the 1D galvo-scanner. For 3D imaging, axial scanning was performed with a 3D translation stage (M-VP-25XA-XYZL, Newport, Bozeman, MT, USA).

### 2.2. The Principle of HiLo-LSTFM

As described in the insert of Figure 1, at the focal plane, the excitation laser is shaped into a spatio-temporal focusing line with Gaussian distribution along the lateral scanning direction (Gaussian modulation line). To obtain 2D images, we scanned the samples with the 1D galvo scanner. Since the size of camera pixel was corresponding to 0.234 μm at the focal plane, we set the scanning interval as 2 camera pixels, which satisfied the Nyquist sampling theorem [33]. To record the 2D images excited by the Gaussian modulation line, we set the camera to the region-of-interest (ROI) sub-arrays mode, as done in extended detection [18].

**Figure 2:**
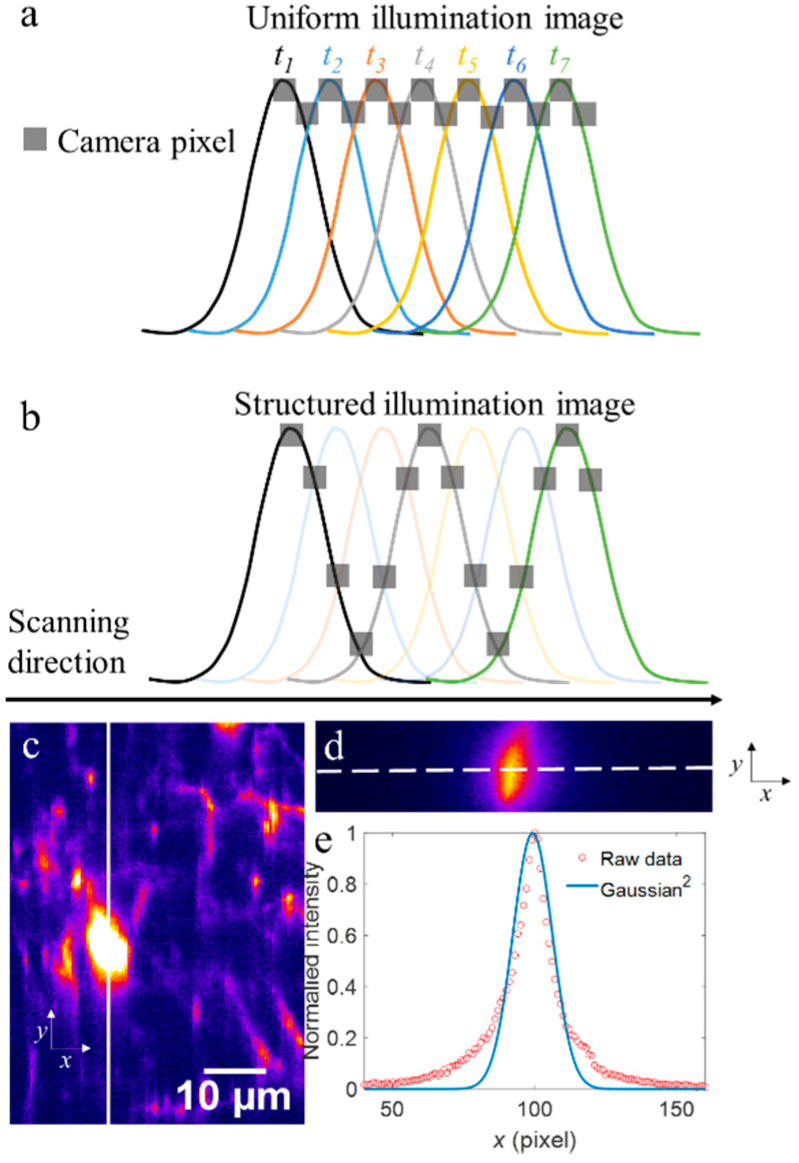
Principle of HiLo-LSTFM. (**a**,**b**) Schematic of sampling in forming uniform illumination image and structured illumination image, respectively. Different colors are used to represent the Gaussian modulation lines at different time points (*t_i_*, *i* = 1, 2, 3…)/positions. Gray squares are used to indicate the sampling of camera pixels. For each time point, the central line and its adjacent lines in an exposure are used to make up the uniform illumination image. Structured-illumination image is formed by interval sampling the sub-images excited by Gaussian lines at different time points/positions. (**c**) The image of neurons in Thy1-YFP mouse, based on virtual confocal slit detection. (**d**) The raw image along the white solid line in (**d**), based on extended detection. (**e**) Profile along the dashed line in (**d**).

Different from conventional HiLo microscopy, the two images required to reconstruct an optical-sectioning image, namely uniform illumination image and structured illumination image (denoted as $I_{u}\left(\vec{\rho }\right)$ and $I_{s}\left(\vec{\rho }\right)$, respectively, where $\vec{\rho }$ is 2D spatial coordinate), were obtained in one-time imaging. We took the Gaussian intensity distribution of illumination line as a naturally structured modulation, as demonstrated in wide-field, single-photon fluorescence microscopy [30,31,32]. As shown in Figure 2, we achieved uniform illumination image and structured illumination image by sampling the camera recorded images in different ways. We used different colors to represent the Gaussian modulation lines at different time points (*t_i_*, *i* = 1, 2, 3…)/positions, and used gray squares to indicate sampling camera pixels. As shown in Figure 2a, at each time point, the central line and its adjacent lines were used to form the uniform illumination image at corresponding spatial positions. Structured illumination image was formed by interval sampling of the Gaussian curve at different time points. Meanwhile, the period of structured pattern could be flexibly customized by changing the sampling interval (Figure 2b).

After obtaining the two images, we extracted the in-focus high-frequency components ($I_{Hi}\left(\vec{\rho }\right)$) by applying a high-pass filter on the uniform illumination image:

(1) $$I_{Hi}(\vec{\rho })=HP[I_{u}(\vec{\rho })]$$

where *HP* indicates Gaussian high-pass filter with the cutoff frequency *Kc*.

To obtain the in-focus low-frequency components, we firstly need to estimate the contrast of structured illumination image:

(2) $$C_{s}(\vec{\rho })=\sigma [I_{s}(\vec{\rho })]$$

where σ  is the standard deviation.

However, to avoid sample induced contrast in practical imaging, we typically use the difference image:

(3) $$I_{d}(\vec{\rho })=I_{u}(\vec{\rho })-I_{s}(\vec{\rho })$$

for contrast reconstruction. Now we can estimate the illumination induced contrast by:

(4) $$C(\vec{\rho })=\sigma [I_{d}(\vec{\rho })]$$

The in-focus low-frequency components then can be constructed by applying a low-pass filter (*LP*) to the weighted uniformly-illuminated image:

(5) $$I_{Lo}(\vec{\rho })=LP[C(\vec{\rho })I_{u}(\vec{\rho })]$$

The final optical-sectioning image is synthesized from the fusion of the above two images, resulting in:

(6) $$I_{HiLo}(\vec{\rho })=I_{Hi}(\vec{\rho })+\eta I_{Lo}(\vec{\rho })$$

where *η* is a scaling factor that ensures a seamless transition at full frequency bandwidth.

It should be noticed that the intensity difference between the central line and its adjacent line in the camera recorded raw image is negligible, which thus will not affect the uniformity of uniform illumination image. However, when deviating from the central line, the intensity difference becomes evident, which ensures a deep modulation depth of the structured illumination image. To verify the claim above, we performed in vivo imaging of neurons in Thy1-YFP mouse, at a depth of 80 µm under the dura. Considering the illumination line is of Gaussian distribution, the two-photon excited fluorescence should follow the distribution of Gaussian square (named as Gaussian^2^ in the following text), which is recorded by operating the camera at a sub-array of 200 lines to perform extended detection [18]. Figure 2c shows the image of neurons based on virtual confocal slit detection [29]. We show the raw image recorded by extended detection in Figure 2d, which is along the solid line in the center of a neural soma (Figure 2c). The profile of raw data along the scanning direction (*x* axis, indicated by the dashed line in Figure 2d) is shown in Figure 2e with red circles, and the Gaussian^2^ fitted curve is also shown as a blue curve. It suggests that the intensity difference between the center and two adjacent values is negligible, and the fitting of Gaussian^2^ distribution agrees well with expectations.

## 3. Results

All procedures involving mice were approved by the Animal Care and Use Committees of Tsinghua University.

### 3.1. Volumetric Imaging of Neurons in Thy1-YFP Mouse Brains In Vivo

**Figure 3:**
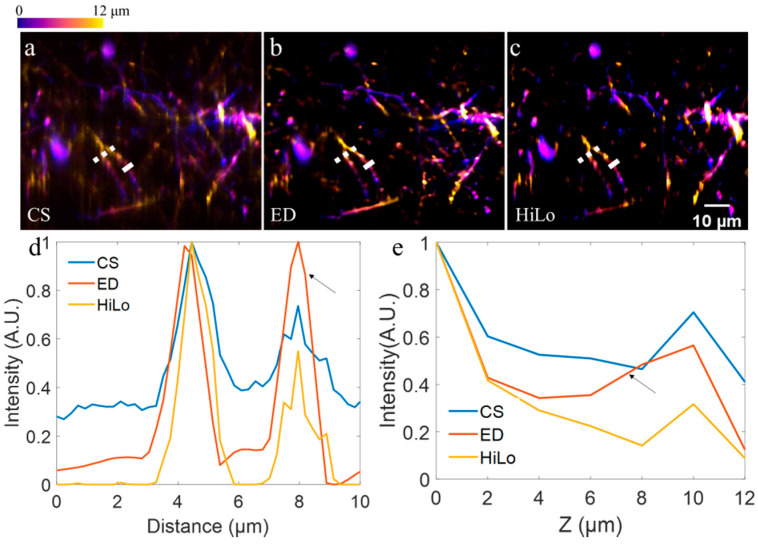
Volumetric imaging of neurons in Thy1-YFP mouse brains. (**a**–**c**) Color-coded axial stack based on virtual confocal slit detection (CS), extended detection (ED), and our proposed method here (HiLo), respectively. (**d**) Profiles along dotted lines in (**a**–**c**), with each profile being normalized. (**e**) Gaussian-fitted peak intensity along solid lines in (**a**–**c**) versus axial plane.

To demonstrate the effectiveness of our method, we first perform volumetric imaging of Thy1-YFP mice (JAX No. 003782) in vivo. After craniotomy, acute imaging of neurons in the cerebral cortex in the living mice under anesthesia were performed, as described in [34]. The imaging depth is 80–92 μm under the dura, and the axial scanning interval was 2 μm. To ensure the fidelity signal to noise ratio, the exposure time for each acquisition was 100 ms. In order to compare with our previously proposed method based on extended detection and computational reconstruction [18], the results with extended detection are also shown. Specifically, in Figure 3a–c, we show color-coded axial stacks based on virtual confocal slit detection (CS), extended detection (ED) [18], and our proposed method (HiLo), respectively. To verify the contrast enhancement of our method, we show profiles along dotted lines (dotted lines in Figure 3a–c, respectively) in Figure 3d. Each profile is normalized to show the contrast. As expected, compared with conventional CS based image, the image based on ED and computational reconstruction shows higher image contrast, resulting from its capability of the scattering-induced noise resistance [18]. However, if we consider CS image as a ground truth, such a deconvolution method in ED method may introduce artifacts, as indicated by the black arrow in Figure 3d. In contrast, this problem does not exist in the HiLo method. From Figure 3d, we can see that the result based on HiLo method not only shows higher contrast, but also maintains the same profile as that in CS image.

We further investigated the axial confinement enhancement of our proposed method. Solid lines in Figure 3a–c are chosen as ROIs. Specifically, for each plane, the Gaussian-fitted peak intensity along the line is set as the ROI intensity distribution at the corresponding plane. We show the intensities versus axial depth in Figure 3e, with each profile being normalized. As shown in Figure 3e, the image based on ED (red line in Figure 3e) shows tighter axial confinement than that of CS method (blue line in Figure 3e). However, the image based on ED also shows different trends (black arrow in Figure 3e), which may indicate artifacts induced by deconvolution. In comparison, the result based on the HiLo method not only shows tighter axial confinement, but also shows the same trend as that in CS image.

### 3.2. Volumetric Imaging of Microglial Cells in CX3CR1-GFP Mouse Brains In Vivo

**Figure 4:**
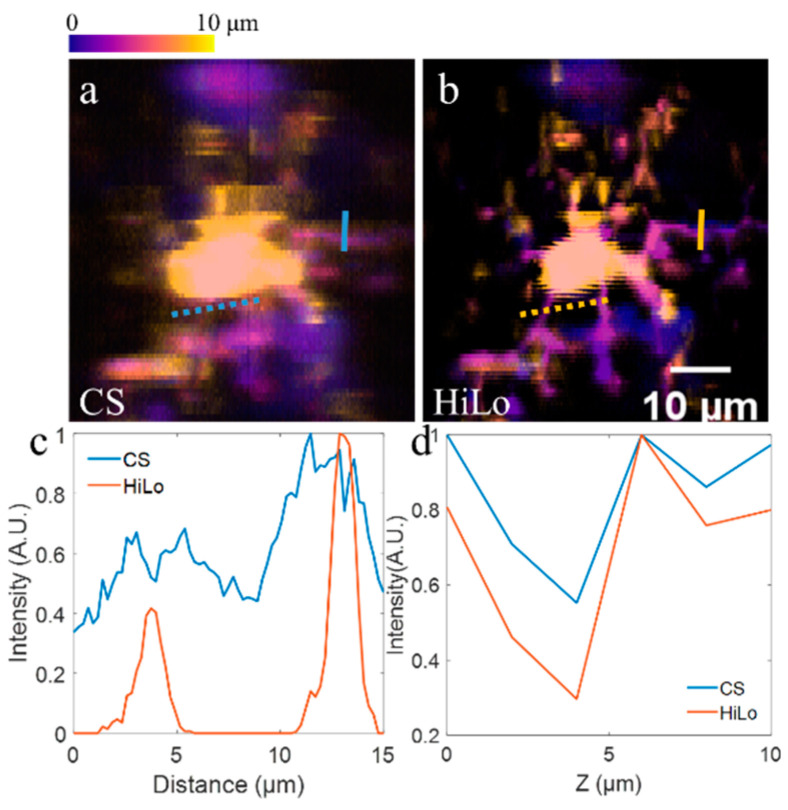
Volumetric imaging of microglia cells in CX3CR1-GFP mouse brains. (**a**–**b**) Depth color coded stack (thickness = 10 μm) based on virtual confocal slit detection (CS) and our proposed HiLo method (HiLo), respectively. (**c**) Profiles of dotted lines in (**a**,**b**), with each profile being normalized. (**d**) Gaussian-fitted peak intensity along solid lines in (**a**–**c**) versus axial plane.

We then perform volumetric imaging of microglia cells in living CX3CR1-GFP mice (JAX No. 005582) in vivo to demonstrate the superiority of our method. The procedure of acute imaging of CX3CR1-GFP mice in vivo is similar as above. The imaging depth is 120–130 μm under the dura, and axial interval is 2 μm. The exposure time for each acquisition is 100 ms. Figure 4a,b show depth-color-coded stacks (thickness = 10 μm) based on virtual confocal slit detection (CS) and our proposed method here (HiLo), respectively. As shown in Figure 4a,b, in the image based on CS, it is difficult to distinguish fine processes of the microglia, while they can be clearly resolved in the image based on HiLo. In order to quantitatively demonstrate the contrast enhancement, we draw dotted lines crossing two processes in Figure 4a,b, and show normalized profiles in Figure 4c. As expected, the profiles of two processes in CS based image (blue line) show noisy contour, suffering from tissue scattering. However, the sharp peaks in the profiles in HiLo-based image (red line) indicate that the processes can be clearly resolved. To investigate the axial confinement, we plot solid lines in Figure 4a,b, and set them as ROIs. For each axial plane, we also set the Gaussian-fitted peak intensity value along the line as the intensity distribution at corresponding plane. We show the intensities versus axial depth in Figure 4d, which suggests tighter axial confinement in our HiLo-based method.

### 3.3. Dynamical Imaging of Microglial Cells in CX3CR1-GFP Mouse Brains In Vivo

**Figure 5:**
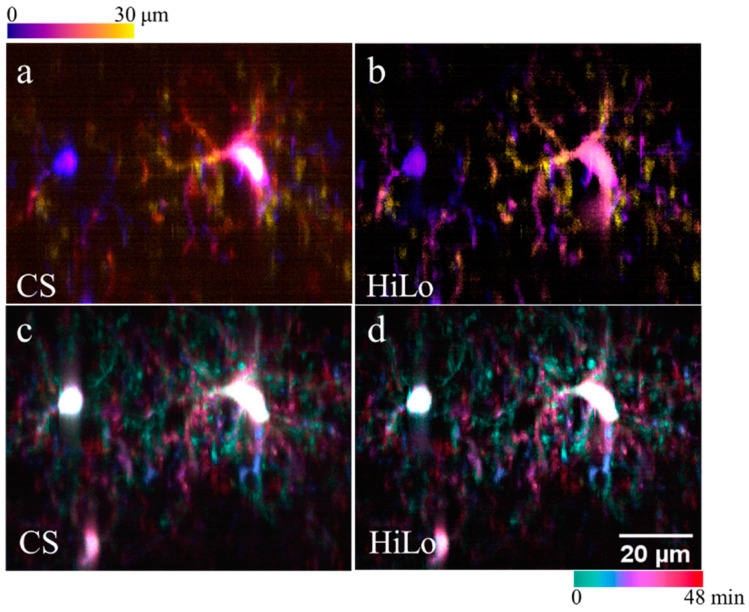
Dynamical imaging of microglia cells in CX3CR1-GFP mouse brains. The whole recording time is 48 min over a volume of 30 μm thickness. (**a**,**b**) Depth color coded stacks based on CS and HiLo methods, respectively. (**c**,**d**) Temporal color-coded maximum-intensity-projections (along axial directions of the image stacks) based on CS and HiLo methods, respectively.

We also performed dynamic imaging of microglial cells in CX3CR1-GFP mouse brains at a depth of 100–130 μm, under the dura. We set the axial interval as 2 μm, and exposure time for each acquisition as 100 ms. Specifically, we recorded the images in a stack of 30 μm thickness over 48 min. In Figure 5a,b, we show depth color coded stacks based on virtual confocal slit detection (CS) and our proposed method here (HiLo), at the end of the time-lapse imaging of 48 min, respectively. Compared with CS based method, the HiLo-based method showed the apparent effectiveness in background suppression. We also show temporal color-coded maximum-intensity-projections (along axial directions of the image stacks) based on CS and HiLo methods in Figure 5c–d, respectively. Again, our HiLo-based method showed the dynamics of fine processes more clearly.

## 4. Discussion and Conclusions

To depress tissue scattering and ensure high temporal resolution in wide-field, optical-sectioning imaging, here we propose a HiLo-based LSTFM, which can enhance the image contrast and axial confinement. We demonstrate the superiority of our method by volumetric imaging in vivo. We compare the contrast and axial confinement of images achieved by the virtual confocal slit detection method, extended detection followed by computational reconstruction method, and our proposed HiLo based method. It shows that the latter two methods can improve image contrast and axial confinement, however, the extended detection method may introduce artifacts in computational reconstruction (deconvolution). To further demonstrate the robustness of our method, we also perform dynamical imaging of microglial cells in CX3CR1-GFP mice.

Different from conventional HiLo microscopy, only one-time imaging is necessary in our HiLo based method. We take the Gaussian intensity distribution of illumination line as a naturally structured modulation; thus, the uniform illumination image and structured illumination image can be achieved by sampling the camera recorded images in different ways. Compared to LSTFM based on extended detection and computational reconstruction, the reconstruction here is more efficient. With a laser of higher pulse energy, the imaging depth can be increased further. We expect its broad applications in high-speed volumetric imaging of biological dynamics.

## Data Availability

Data are available upon reasonable request.

## Abbreviations

LSTFM	Line scanning temporal focusing microscopy
SLM	Spatial light modulators
LSTPM	Line scanning two-photon microscopy
AO	Adaptive optics
SIM-OS	Structured illumination based optical-sectioning microscopy
HiLo	“Hi” and “Lo” for the high and low spatial frequency components, respectively
